# Sedentary Behavior and Light Physical Activity Are Associated with Brachial and Central Blood Pressure in Hypertensive Patients

**DOI:** 10.1371/journal.pone.0146078

**Published:** 2015-12-30

**Authors:** Aline M. Gerage, Tania R. B. Benedetti, Breno Q. Farah, Fábio da S. Santana, David Ohara, Lars B. Andersen, Raphael M. Ritti-Dias

**Affiliations:** 1 Department of Physical Education, Federal University of Santa Catarina, Florianópolis, Santa Catarina, Brazil; 2 Superior School of Physical Education, University of Pernambuco, Recife, Pernambuco, Brazil; 3 Metabolism, Nutrition and Exercise Research Group, Londrina State University, Londrina, Paraná, Brazil; 4 Department of Exercise Sciences and Clinical Biomechanics, University of Southern Denmark, Odense, Denmark; 5 Sogn and Fjordane University College, Sogndal, Norway; 6 Hospital Israelita Albert Einstein, São Paulo, São Paulo, Brazil; Tel Aviv Sourasky Medical Center, ISRAEL

## Abstract

**Background:**

Physical activity is recommended as a part of a comprehensive lifestyle approach in the treatment of hypertension, but there is a lack of data about the relationship between different intensities of physical activity and cardiovascular parameters in hypertensive patients. The purpose of this study was to investigate the association between the time spent in physical activities of different intensities and blood pressure levels, arterial stiffness and autonomic modulation in hypertensive patients.

**Methods:**

In this cross-sectional study, 87 hypertensive patients (57.5 ± 9.9 years of age) had their physical activity assessed over a 7 day period using an accelerometer and the time spent in sedentary activities, light physical activities, moderate physical activities and moderate-to-vigorous physical activities was obtained. The primary outcomes were brachial and central blood pressure. Arterial stiffness parameters (augmentation index and pulse wave velocity) and cardiac autonomic modulation (sympathetic and parasympathetic modulation in the heart) were also obtained as secondary outcomes.

**Results:**

Sedentary activities and light physical activities were positively and inversely associated, respectively, with brachial systolic (r = 0.56; *P* < 0.01), central systolic (r = 0.51; *P* < 0.05), brachial diastolic (r = 0.45; *P* < 0.01) and central diastolic (r = 0.42; *P* < 0.05) blood pressures, after adjustment for sex, age, trunk fat, number of antihypertensive drugs, accelerometer wear time and moderate-to-vigorous physical activities. Arterial stiffness parameters and cardiac autonomic modulation were not associated with the time spent in sedentary activities and in light physical activities (*P* > 0.05).

**Conclusion:**

Lower time spent in sedentary activities and higher time spent in light physical activities are associated with lower blood pressure, without affecting arterial stiffness and cardiac autonomic modulation in hypertensive patients.

## Introduction

Hypertension affects 30 to 45% of adults worldwide[1], and has been associated with stroke, ischemic heart disease and other cardiovascular diseases[2], being responsible for approximately 13% of all deaths worldwide[3].

As part of a comprehensive lifestyle modification approach, hypertensive patients are advised to practice at least 30 min of moderate-to-vigorous physical activity (MVPA) on five to seven days per week[1, 4, 5]. Interventional studies have shown beneficial effects of different structured and supervised exercise interventions on cardiovascular variables[6–9]. However, the influence of the amount and intensity of unsupervised daily physical activities on blood pressure (BP), arterial stiffness—considered as a strong predictor of increased cardiovascular risk[10]—and in heart rate variability[11] have not been sufficiently studied.

Studies have shown controversial results on the influence of the time spent doing MVPA, light physical activities (LPA) and sedentary activities (SED) on cardiovascular health[12–16]. More time spent in MVPA has been associated with lower levels of BP[14] and with some, but not all, indexes of arterial stiffness[14–16]. Additionally, studies have shown that the time spent in LPA was associated with lower BP levels[12] and arterial stiffness[12, 16], but these are not universal findings[13, 15]. Another study has indicated that time spent in SED is inversely related with arterial stiffness parameters[15], while other researchers reported no significant relationships[16]. Interestingly, the single study[14] that analyzed hypertensive subjects observed that lower time spent in SED and higher time spent in LPA are associated with lower BP and arterial stiffness indicators, suggesting that different physical activity intensities have the potential to affect cardiovascular parameters in hypertensive patients. However, since this study did not adjust the regressions for the amount of time spent in MVPA, which has been shown to influence cardiovascular parameters[16], studies using this adjustment are needed to confirm these results.

Understanding of how the time spent in physical activity of different intensities affects cardiovascular parameters in patients with hypertension is necessary to expand public health recommendations for these patients, including suggestions related to physical activity intensity. Thus, the purpose of this study was to investigate the association between the time spent in physical activity of different intensities and BP levels, arterial stiffness and autonomic modulation in hypertensive patients. Our hypothesis was that less time spent in SED and more time spent in physical activities are associated with better BP, arterial stiffness and cardiac autonomic modulation indicators in hypertensive patients.

## Methods

### Sample

#### Recruitment

Patients were recruited for possible enrolment into a Randomized Clinical Trial (NCT02257268) related to a lifestyle modification program in hypertensive subjects. The data and analyses for the current cross-sectional study were part of the baseline assessments obtained for this Randomized Clinical Trial. The recruitment was carried out through local media advertisements and flyers distributed in hospitals and in the surrounding area of the University of Pernambuco, Recife, Pernambuco State (northeast of Brazil), in 2014. The study protocol was approved by the ethics committee of the Federal University of Santa Catarina (811.266) in compliance with the Brazilian National Research Ethics System Guidelines. Written informed consent was obtained from each patient prior to investigation.

#### Screening

As inclusion criteria, participants were required to be 40+ years old, hypertensive and to have been taking antihypertensive drugs for at least three months prior to the study. Additionally, participants were required not to have diabetes, other cardiovascular diseases or physical disabilities, and not to be involved in regular physical activity programs.

### Physical activity assessment

Physical activity was assessed by a GT3X or GT3X+ accelerometer (ActiGraph, Pensacola, FL, USA) and Actilife software (ActiGraph, Pensacola, FL, USA) was used to analyze collected data. Each participant was instructed to use the accelerometer for seven consecutive days, removing it only to sleep, bathe or perform activities involving water. The device was attached to an elastic belt and fixed to the right side of the hip. Data were collected with a 30 Hz sample frequency and were analyzed using 60 s epochs. Periods with consecutive values of zero (with a 2 min spike tolerance) for 60 min or longer were interpreted as “accelerometer not worn” and excluded from the analysis[17]. Physical activity data were included only if the participant had accumulated a minimum of 10 hours/day of recording for at least four days including one weekend day. The average time spent in each physical activity intensity was calculated using the cutoff points proposed by Freedson et al[18], considering SED as 0–99 counts/min, LPA as 100–1951 counts/min, moderate physical activity (MPA) as ≥ 1952 counts/min and vigorous/very vigorous physical activity as ≥ 5725 counts/min using the vertical axis, and analyzed in min/day, adjusting for the number of days the device was worn.

### Outcome measurements

Prior to all outcome measurements, the patients were instructed to avoid physical exercise for at least 24 hours prior to the visit, avoid smoking, alcohol and caffeine ingestion for at least 12 hours and to eat a light meal before arriving at the laboratory. In the laboratory, a rest period of 10 min in the supine position prior to taking the measurements was instructed. All measurements were taken in the supine position in a quiet environment, with monitored temperature. The volunteers were asked to attend the laboratory twice. During the first visit the patients were submitted to anthropometry, heart rate variability and body composition assessments. At the end of this first visit, they received the accelerometer to use during the following seven days. After this period, they returned with the accelerometer to the laboratory for the second visit, at the same time of the day as the first visit and brachial and central BP and arterial stiffness were evaluated, in this order.

### Primary outcome measures

#### Blood pressure

Brachial systolic and diastolic BP were measured on the left arm using an automatic oscillometric instrument (Omron HEM 742-E, Bannockburn, USA). Measurements were taken on two non-consecutive days, and three measurements were performed on each day, with a one minute interval between measurements. The mean of all BP values measured was used for analysis. All the measurements were taken by the same evaluator (systolic BP: ICC = 0.85 and diastolic BP: ICC = 0.92).

Central systolic and diastolic BP were obtained by the pulse wave analysis that was recorded in the left radial artery using applanation tonometry (SphygmoCor—AtcorMedical, Sydney, Australia) and the validated transfer function algorithm provided by the Sphygmocor® software. All measurements were performed by the same evaluator (systolic BP: ICC = 0.84 and diastolic BP: ICC = 0.72), according to guidelines specified by the Clinical Application of Arterial stiffness, Task Force III[19]. To enhance the accuracy of measurements, only those values whose quality index exceeded 80% were used.

### Secondary Outcome Measures

#### Anthropometry, demography, and use of antihypertensive drugs

Body mass was measured with participants wearing light clothes and barefoot, using an automatic scale (Welmy, São Paulo, Brazil) accurate to the nearest 0.1 kg. Height was measured using a stadiometer connected to a scale accurate to the nearest 0.5 cm. Demographic information and a list of current antihypertensive drugs used were obtained by individual interview.

#### Body composition

Total body fat and trunk fat were estimated by densitometry scans for dual-energy X-ray absorptiometry (Lunar Prodigy DXA, model NRL 41990, GE Lunar, Madison, WI). Scans were performed with patients in the supine position along the longitudinal centerline axis of the table. The software generated standard lines that separated the limbs from the trunk and head. For the assessment, participants were instructed to remain clothed, but were asked to remove any metallic objects. The procedure lasted five to ten minutes for each individual and was carried out by the same technician who calibrated the device. All the procedures were carried out following the manufacturer’s recommendations. The percentage of fat was calculated by dividing the amount of fat by the weight of the segment analyzed (trunk or all body).

#### Arterial stiffness and wave reflection parameters

The arterial stiffness and wave reflection parameters were obtained through carotid-femoral pulse wave velocity (cfPWV) and augmentation index respectively. For these measurements, the applanation tonometry (SphygmoCor—AtcorMedical, Sydney, Australia) method was used. The measurement of these parameters was performed by the same evaluator (cfPWV: ICC = 0.91 and AI: ICC = 0.80) according to guidelines specified by the Clinical Application of Arterial Stiffness, Task Force III[19]. The augmentation index was expressed as a percentage of the ratio of augmented pressure to pulse pressure, based on the pulse wave analysis measured in the left radial artery. For measurement of cfPWV, the sternal notch to carotid distance was subtracted from the total distance between carotid and femoral. Simultaneous ECG was assessed to obtain heart rate and, according to a “foot-to-foot” method, the time difference between the points was measured. Then, the distance between the two arteries (D) was divided by the time difference (Δt). Thus, the PWV = D/(Δt) (m/s).

#### Cardiac autonomic modulation

For cardiac autonomic modulation assessment, R-R interval was obtained using a heart rate monitor (Polar, RS 800CX; Polar Electro Oy Inc, Kempele, Finland). Participants remained in the supine position for 10 minutes, after approximately 10 minutes at rest. All analyses were performed with Kubios HRV software (Biosignal Analysis and Medical Imaging Group, Joensuu, Finland) by a single evaluator (ICC = 0.99), following the recommendations of the Task Force of the European Society of Cardiology and the North American Society of Pacing and Electrophysiology[20]. The frequency-domain parameters were analyzed using spectral analysis of heart rate variability. Stationary periods of the tachogram of at least 5 min were broken down into bands of low (LF) and high (HF) frequency using the autoregressive method with a fixed model order of 12 according to Akaike’s information criteria. Frequencies between 0.04 and 0.4 Hz were considered physiologically significant; the LF component was represented by oscillations between 0.04 and 0.15 Hz, and HF was represented by oscillations between 0.15 and 0.4 Hz. The power of each spectral component was normalized by dividing the power of each spectrum band by the total variance, minus the value of the very low frequency band (<0.04 Hz), and multiplying the result by 100[20]. To interpret the results, the LF and HF of the heart rate variability, showed in normalized units (n.u.), were considered, respectively, as markers of predominantly sympathetic and parasympathetic modulation of the heart[20].

### Statistical analyses

The data were stored and analyzed using the Statistical Package for the Social Sciences (SPSS Version 17.0 for Windows). Normality was checked using the Shapiro-Wilk test and the Levene test was used to analyze the homogeneity of variances. Continuous variables were summarized as mean and standard deviations or in median and inter-quartile range, whereas categorical variables were summarized as relative frequencies.

Mean values of cardiovascular risk factors were compared using the ANCOVA one-way test with sex, age, trunk fat, number of antihypertensive drugs, MVPA and accelerometer wear time as covariables, according to the level of physical activity, categorized by low, moderate and high, according to tertiles, for SED (1^st^ tertile: < 492.42; 2^nd^ tertile: 492.42–570.67; 3^rd^ tertile: > 570.67 min/day) and LPA (1^st^ tertile: < 297.54; 2^nd^ tertile: 297.54–356.86; 3^rd^ tertile: >356.86 min/day). Comparison among patients classified according to tertiles of minutes spent in MPA (1^st^ tertile: < 12.7; 2^nd^ tertile: 12.7–23.1; 3^rd^ tertile: > 23.1 min/day) was also tested by ANCOVA one-way, with sex, age, trunk fat, number of antihypertensive drugs, accelerometer wear time and LPA as covariables.

Multiple linear regression analyses were conducted to examine the relationship between SED and LPA and cardiovascular parameters, adjusting for sex, age, trunk fat, number of antihypertensive drugs used and accelerometer wear time (model 1) and also including MVPA (model 2). The same approach was employed to analyze the relationship between MPA and cardiovascular parameters adjusting for sex, age, trunk fat, number of antihypertensive drugs used and accelerometer wear time with and without including LPA. A residual analysis was performed, and adherence to the normal distribution was tested using the Shapiro-Wilk test. Multicollinearity analysis was performed assuming variance inflation factors less than five and tolerance below 0.20. For all the statistical analyses, significance was accepted at *P* < 0.05.

The required sample size (n = 51) for linear multiple regression test was calculated using the software GPower (3.1.9), considering the brachial systolic BP as the main variable, an alpha of 95%, a power of 80%, an effect size of 0.33 predicted by a squared correlation coefficient of 0.25, and seven variable predictors.

## Results

Eighty-seven patients (57.5 ± 9.9 years old; 79% female) participated in this study (Table 1). Minutes spent in SED, LPA, and MVPA accounted for 60 ± 9%, 37 ± 8% and 3 ± 3% of daily physical activity, respectively.

**Table 1:** General characteristics, physical activity and cardiovascular risk factors information of participants (n = 87).

Variable	Mean (SD)
Age (years)*	55 (51–64)
Weight (kg)*	76.7 (68.2–87.3)
Height (cm)*	158.0 (154.0–163.0)
Body mass index (kg/m^2^)	31.1 (5.5)
Total body fat (%)*	43.8 (38.1–46.5)
Trunk fat (%)*	43.8 (39.1–47.0)
Number of antihypertensive drugs	2.0 (0.9)
bSBP (mmHg)	133.0 (16.5)
bDBP (mmHg)	80.1 (8.7)
cSBP (mmHg)	126.2 (17.2)
cDBP (mmHg)	81.5 (9.4)
Augmentation index (%)	31.6 (10.0)
Pulse wave velocity (m/s)^††^	10.2 (2.2)
Low frequency (n.u.)	52.4 (20.4)
High frequency (n.u.)	47.6 (20.4)
Sedentary time (min/day)	531.8 (95.2)
Light physical activity (min/day)	329.4 (83.3)
Moderate physical activity (min/day)	24.5 ± 22.7
Vigorous physical activity (min/day)	0.2 ± 0.9
Wear time (min/day)	885.8 (88.5)
Days worn (days/week)	6.3 (0.9)

bSBP = brachial systolic blood pressure; bDBP = brachial diastolic blood pressure; cSBP = central systolic blood pressure; cDBP = central diastolic blood pressure.

*Expressed as median (inter-quartile range).

^††^ n = 55.

**Fig 1:**
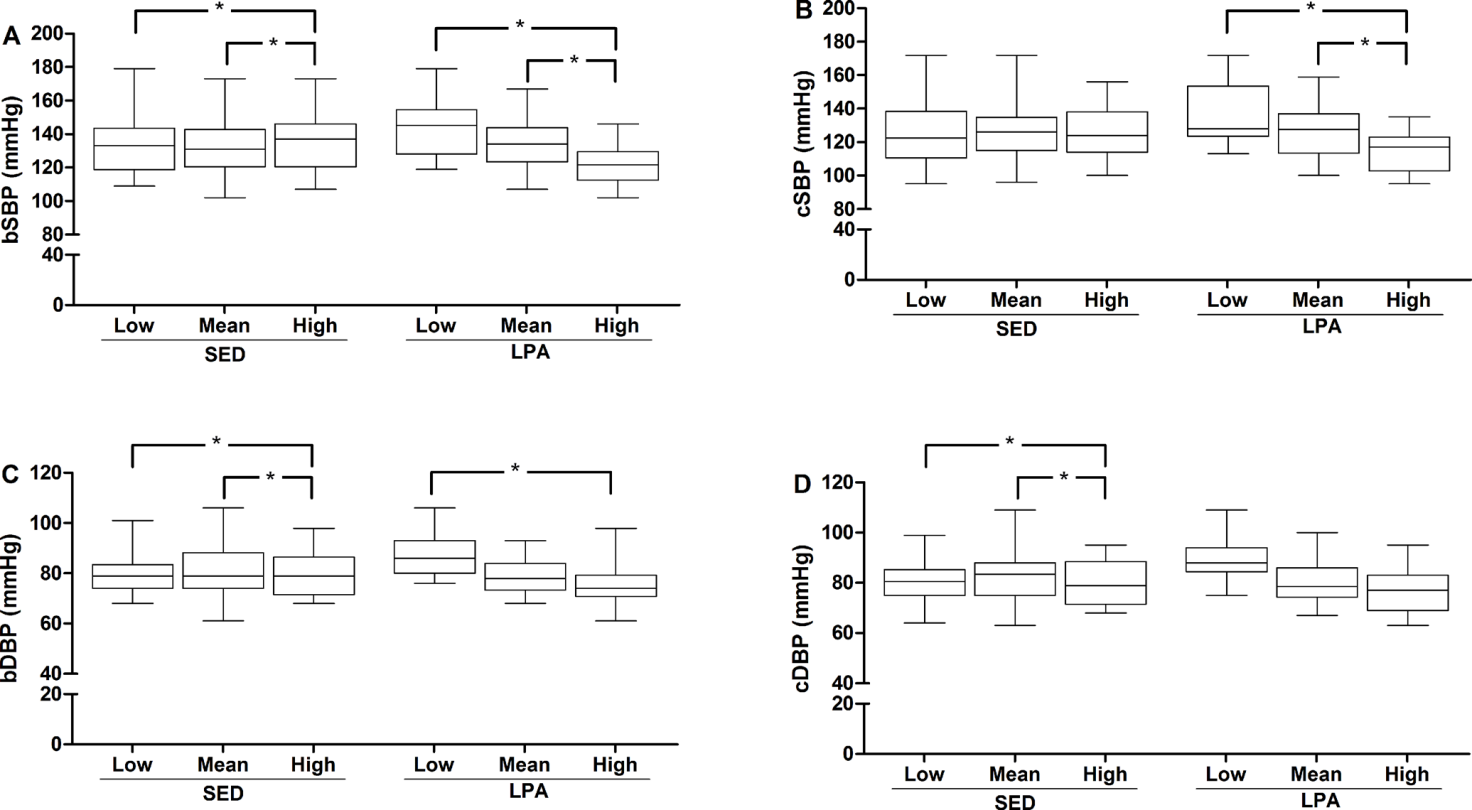
Comparison of blood pressure among tertiles of minutes spent in sedentary activities and in light physical activities. bSBP = brachial systolic blood pressure; bDBP = brachial diastolic blood pressure; cSBP = central systolic blood pressure; cDBP = central diastolic blood pressure. For sedentary activities, low is < 492.42; mean is 492.42–570.67; and high is > 570.67 min/day. For light physical activities, low is < 297.54; mean is 297.54–356.86; and high is >356.86 min/day. *Note*: All analyses were adjusted for sex, age, trunk fat, number of antihypertensive drugs, accelerometer wear time and moderate-to-vigorous physical activities.

After adjustments it was observed that the patients with higher time in SED presented higher systolic (brachial) and diastolic (brachial and central) BP (*P* < 0.05), while the group with higher time in LPA presented lower systolic (brachial and central) and diastolic (brachial) BP (*P* < 0.05) (**Fig 1**).

There were no differences in arterial stiffness and in cardiac autonomic parameters among patients classified according to tertiles of minutes spent in SED or LPA (*P* > 0.05) (Table 2).

**Table 2:** Comparison of arterial stiffness and cardiac autonomic modulation among tertiles of minutes spent in sedentary activities and light physical activities.

	Minutes per day in sedentary activities			Minutes per day in light physical activities		
	Low(< 492.42)	Mean(492.42–570.67)	High(> 570.67)	Low(< 297.54)	Mean(297.54–356.86)	High(>356.86)
AI (%)	32.8 ± 11.5	29.8 ± 10.2	32.3 ± 8.0	30.2 ± 10.3	32.4 ± 11.4	32.3 ± 8.2
PWV (m/s) ^††^	10.1 ± 1.5	9.9 ± 2.1	10.5 ± 2.8	10.6 ± 3.1	10.2 ± 1.9	9.9 ± 1.6
LF (n.u)	47.9 ± 20.9	53.8 ± 20.3	54.2 ± 20.5	54.1 ± 22.0	51.9 ± 19.3	51.3 ± 20.4
HF (n.u.)	48.0 ± 20.1	54.9 ± 21.3	54.2 ± 19.8	45.9 ± 22.0	48.2 ± 19.3	48.7 ± 20.4

Low = 1^st^ tertile; Mean = 2^nd^ tertile; high = 3^rd^ tertile; AI = augmentation index; PWV = pulse wave velocity; LF = low-frequency; HF = high frequency.

^††^ n = 55.

Regarding the comparison among patients classified according tertiles of minutes spent in MPA, significant differences were observed only for LFnu and HFnu (F = 4.59; *P* = 0.01). Those classified in the first tertile (< 12.7 min/day of MPA) had LF and HF higher and lower (Δ = 7.5 (n.u.)), respectively, than those in the 3^rd^ tertile (> 23.1 min/day of MPA). Considering MVPA, the results of this analysis were similar.

Table 3 shows the association between LPA and BP, arterial stiffness and cardiac autonomic modulation.

**Table 3:** Relationship between light physical activities and blood pressure, arterial stiffness and cardiac autonomic modulation parameters in hypertensive patients.

	Model 1*		Model 2**	
	ß (95% CI)	*P*	ß (95% CI)	*P*
bSBP (mmHg)	-0.059 (-0.102; -0.015)	0.009	-0.068 (-0.113; -0.023)	0.003
bDBP (mmHg)	-0.035 (-0.059; -0.010)	0.006	-0.036 (-0.061; -0.010)	0.007
cSBP (mmHg)	-0.051 (-0.098; -0.004)	0.032	-0.054 (-0.103; -0.006)	0.029
cDBP (mmHg)	-0.032 (-0.059; -0.005)	0.022	-0.031 (-0.059; -0.002)	0.033
AI (%)	0.001 (-0.028; 0.030)	0.941	0.005 (-0.025; 0.035)	0.730
PWV (m/s)^††^	-0.002 (-0.009; 0.004)	0.461	-0.002 (-0.009; 0.005)	0.497
LF (n.u.)	-0.049 (-0.104; 0.006)	0.080	-0.038 (-0.094; 0.018)	0.177
HF (n.u.)	0.049 (-0.006; 0.104)	0.080	0.038 (-0.018; 0.094)	0.177

ß (95% CI) = Regression coefficient (95% confidence interval); bSBP = brachial systolic blood pressure; bDBP = brachial diastolic blood pressure; cSBP = central systolic blood pressure; cDBP = central diastolic blood pressure; PWV = pulse wave velocity; LF = low frequency; HF = high frequency.

^††^ n = 55.

*Adjusted for sex, age, trunk fat, number of antihypertensive drugs and accelerometer wear time.

**Adjusted for sex, age, trunk fat, number of antihypertensive drugs, accelerometer wear time and moderate-to-vigorous physical activities.

**Fig 2:**
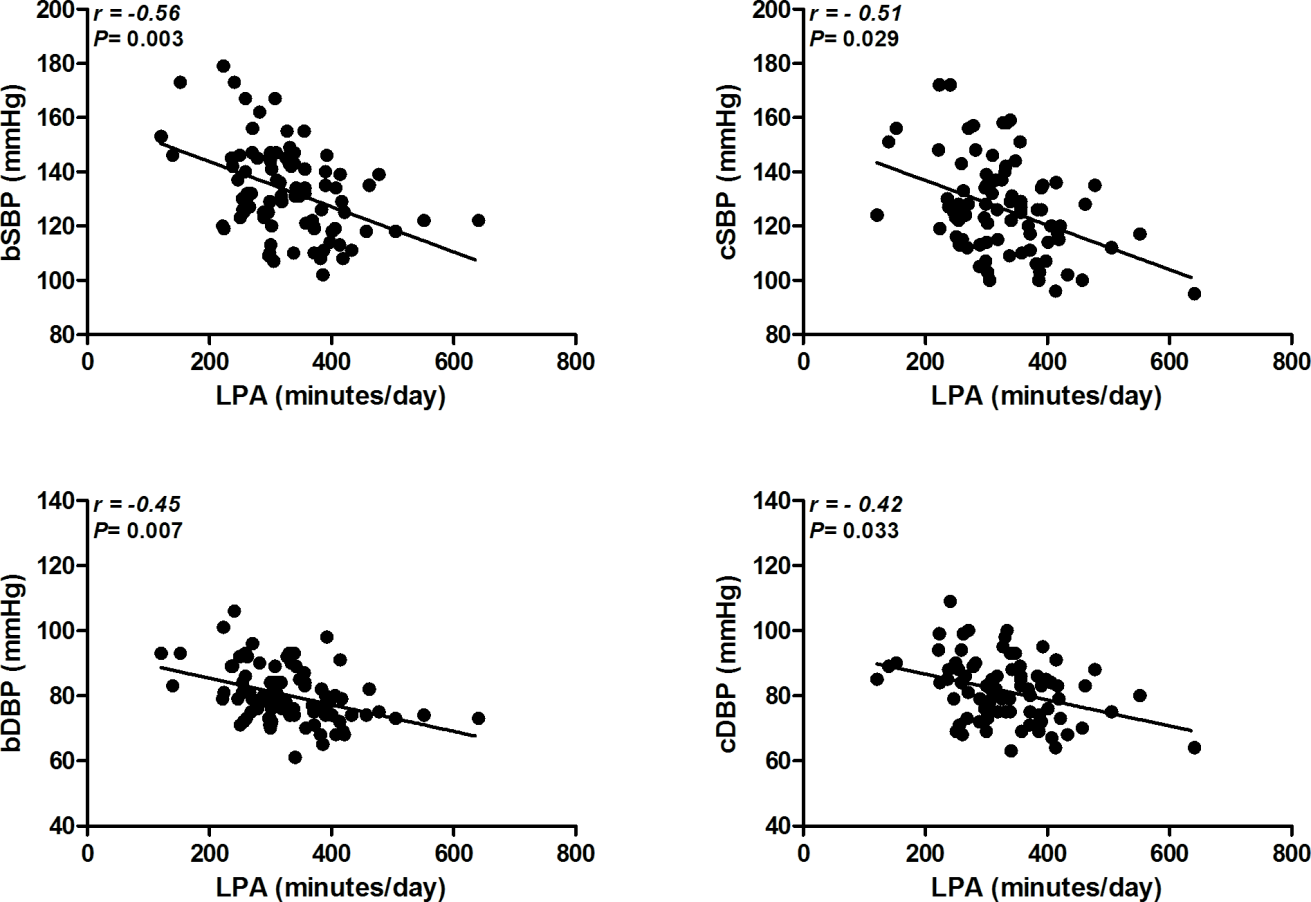
Relationship between light physical activity and brachial and central blood pressure. bSBP = brachial systolic blood pressure; bDBP = brachial diastolic blood pressure; cSBP = central systolic blood pressure; cDBP = central diastolic blood pressure; LPA = light physical activities. *Note*: All analyses were adjusted for sex, age, trunk fat, number of antihypertensive drugs, accelerometer wear time and moderate-to-vigorous physical activities.

Inverse relationships were observed between LPA and BP (brachial and central systolic and diastolic) (*P* < 0.05) after adjustment for sex, age, trunk fat, number of antihypertensive drugs and accelerometer wear time (model 1). When MVPA was included in the adjusted analysis (model 2) the relationships between LPA and all the BP measurements remained significant (*P* < 0.05) (**Fig 2**). Arterial stiffness parameters and cardiac autonomic modulation were not related to LPA in both models of adjusted analysis (*P* > 0.05).

Inversely to results seen with LPA, time spent in SED was positively associated with brachial and central systolic and diastolic BP both in model 1 (ß = 0.44 to 0.29; *P* < 0.05) and model 2 (ß = 0.68 to 0.31; *P* < 0.05). There were no relationships between SED and arterial stiffness and cardiac autonomic modulation (*P* > 0.05).

Time spent in MPA was not associated with BP (bSBP: ß = 0.07 and *P* = 0.43; bDBP: ß = -0.02 and *P* = 0.67; cSBP: ß = -0.01 and *P* = 0.88; cDBP: ß = -0.05 and *P* = 0.36), arterial stiffness (AI: ß = -0.06 and *P* = 0.30; PWV: ß = -0.003 and *P* = 0.79) or cardiac autonomic modulation (LF: ß = -0.20 and *P* = 0.06; HF: ß = 0.20 and *P* = 0.06) after adjustment for confounders.

No significant association was observed between SED, LPA or MPA and the number of antihypertensive drugs used (*P* > 0.05), before or after adjustment for confounders.

## Discussion

The main findings of the present study were: (i) SED and LPA were related to both brachial and central BPs in hypertensive patients; (ii) arterial stiffness and cardiac autonomic modulation parameters were not associated with the time spent in SED and LPA. The BP results concurred with our hypothesis, while the findings regarding arterial stiffness and cardiac autonomic modulation parameters did not.

The strengths of the study include the use of scientifically recognized techniques to assess physical activity, brachial and central BPs. Brachial BP was measured twice in non-consecutive days and only hypertensive patients were included in the study, contributing to a better comprehension about the influence of different physical activity intensities on hypertension. In addition the inclusion of the trunk fat as a confounder factor should be highlighted, since it was assessed by one of the best techniques to evaluate body composition[21], and because obesity has been considered the major risk factor for the development of hypertension. Increased adiposity, especially visceral fat, activates the renin-angiotensin-aldosterone system and the sympathetic nervous system in addition to a physical compression of the kidneys, leading to altered intrarenal hemodynamics and impaired sodium excretion, which contribute to increased BP and to a higher difficulty for BP control[22].

The relationship between the time spent in SED and BP observed in this study is in agreement with a previous study of hypertensive patients[14]. Additionally, the literature has shown a positive relationship between SED and clustered metabolic syndrome risk score[13] and mortality[23]. Interestingly, in our study the relationships remained significant after adjustments for trunk fat and MVPA, which could confound this relationship. The mechanisms underlying the relationship between SED and BP are not well understood, however it has been shown that decreases in skeletal muscle contraction due to prolonged time spent in SED suppress the lipoprotein lipase action, increasing free radical production and inflammation and consequently increasing BP[24]. Nevertheless, future studies are required for a better understanding of the biological link between the time spent in SED and BP.

Associations between higher time spent in LPA and improvements in several cardiovascular risk factors such as triglycerides[25], HDL cholesterol[13, 26], and waist circumference[13, 26] have been previously reported in healthy patients[25] and in subjects with metabolic syndrome[13, 26]. This study showed that higher time spent in LPA is also associated with lower BP levels in hypertensive patients, which is in agreement with previous studies involving healthy adults[12] and patients with hypertension[14], although some researchers did not find the same results[13]. In practical terms, the results of linear regression indicated that each 100 minutes per day engaging in LPA is associated with a decrease of 6.8 and 3.6 mmHg in brachial systolic and diastolic BPs, respectively. As a 2 mmHg reduction of systolic BP results in a 6% decrease in stroke mortality and a 4% decrease in mortality attributable to coronary heart disease[27], these findings potentially impact the morbidity of hypertensive patients.

Interestingly, LPA was also inversely associated with central BP, indicating that LPA influences both peripheral and central arteries. Hypertensive patients present increased BP in both central and peripheral arteries, however antihypertensive treatment strategies have shown different effects on brachial and central BP, most of them acting mainly in peripheral arteries[28, 29]. As central BP has been considered a strong predictor of cardiovascular events and target organ damage[30], the beneficial association observed between LPA and a reduction in central BP indicates a possible effect of physical activity, even in low intensity, in reducing cardiovascular risk in hypertensive patients. The mechanisms linking LPA and decreases in central BP are not clear. However, considering that central BP has been related with peripheral vasculature[31], we speculate that there is a peripheral effect of LPA[32], which should be further investigated.

The time spent in MPA was not associated with BP. Although these results are in disagreement with previous studies[14, 33] which showed inverse relationships between MVPA and BP levels, it is important to highlight that our patients performed less than 1 min/day of vigorous physical activity. As the time spent in moderate and vigorous physical activities has not been described, it is possible that in previous studies subjects spent more time engaging in vigorous activity, which could explain the observed associations. In fact, there is evidence[34] that daily physical activity duration, rather than intensity, is associated with cardiovascular risk factors in older adults not engaged in exercise programs. Therefore, the lack of association between MPA and BP may also be related to the short time (low volume) spent doing this type of activity by patients in this study.

In addition neither SED, LPA nor MPA were associated to arterial stiffness parameters and cardiac autonomic modulation, which does not concur with previous studies[12, 14, 35]. As the present study included only subjects not engaged in regular exercise programs and taking antihypertensive medication, the controversy between studies may be caused by the different types of physical activity practiced and drugs used. Our results suggest that time spent in SED, daily low intensity physical activities and unstructured MPA (or MVPA) are not sufficient to promote benefits in these cardiovascular components. In fact, improvements in arterial stiffness[7, 36] and in heart rate variability[37] have been observed after regular high or moderate intensity exercise training programs. Thus, a regular training program with adequate exercise intensity may be necessary to improve arterial stiffness and heart rate variability, instead of sporadic daily physical activities. Additionally, the influence of medication use on these results cannot be ignored, considering that other studies[12, 14] included non-medicated subjects.

The positive and inverse relationship between SED and LPA, respectively, and brachial and central BP show the importance of LPA in hypertensive patients. Thus, physical activity prescription should consider the recommendation of LPA for BP control, especially because it is easier to include in daily activities[24]. Therefore, we suggest that the physical activity recommendations for hypertensive patients should emphasize the importance of replacing SED with LPA. Simple changes in daily routine, such as using the stairs instead of the elevator, walking instead of using the car for short distances, reducing screen time, and breaking long periods of sitting time with movement could provide benefit to these patients.

The cross-sectional design is the major limitation of this study. Interventional studies are required to confirm these findings and to make inferences about causality. Additionally, we suggest for future studies the rigorous control of antihypertensive drugs. The patients were using different antihypertensive drugs. Although the lack of association between SED, LPA or MPA and the number of antihypertensive drugs used may be related to other factors related to cardiovascular control (i.e. genetic, hormones, eating habits, other pathologies), specific influence of the type and dose can have occurred, which was not controlled in the study. The small sample size and the impossibility to access cfPWV of all participants due to technical difficulties, should also be considered as limitations of this study, and could partially help to explain why the expected association between SED and LPA and arterial stiffness and cardiac autonomic modulation was not seen. Finally, as the subjects of this study were not engaged in regular exercise programs, the results could not be extrapolated for patients who are engaged in such programs.

In conclusion, this study indicated that lower time spent in SED and higher time spent in LPA are associated with lower brachial and central BP, without affecting arterial stiffness and autonomic modulation in hypertensive patients.

## Supporting Information

S1 Fig Comparison of blood pressure among to tertiles of minutes spent in sedentary activities and in light physical activities. SED = sedentary activities; LPA = light physical activities; bSBP = brachial systolic blood pressure; bDBP = brachial diastolic blood pressure; cSBP = central systolic blood pressure; cDBP = central diastolic blood pressure.
(XLS)

S2 Fig Relationship between light physical activity and brachial and central blood pressure. LPA = light physical activities; bSBP = brachial systolic blood pressure; bDBP = brachial diastolic blood pressure; cSBP = central systolic blood pressure; cDBP = central diastolic blood pressure.
(XLS)

## References

[1] Mancia G, Fagard R, Narkiewicz K, Redon J, Zanchetti A, Bohm M, et al 2013 ESH/ESC Guidelines for the management of arterial hypertension: The Task Force for the management of arterial hypertension of the European Society of Hypertension (ESH) and of the European Society of Cardiology (ESC). Eur Heart J. 2013;34(28):2159–219. 10.1093/eurheartj/eht151 . 23771844

[2] Lawes CM, Vander Hoorn S, Rodgers A, International Society of H. Global burden of blood-pressure-related disease, 2001. Lancet. 2008;371(9623):1513–8. 10.1016/S0140-6736(08)60655-8 . 18456100

[3] Lewington S, Clarke R, Qizilbash N, Peto R, Collins R. Age-specific relevance of usual blood pressure to vascular mortality: a meta-analysis of individual data for one million adults in 61 prospective studies. Lancet. 2002;360(9349):1903–13. . 12493255 10.1016/s0140-6736(02)11911-8

[4] Pescatello LS, Franklin BA, Fagard R, Farquhar WB, Kelley GA, Ray CA. American College of Sports Medicine position stand. Exercise and hypertension. Med Sci Sports Exerc. 2004;36(3):533–53. . 15076798 10.1249/01.mss.0000115224.88514.3a

[5] Eckel RH, Jakicic JM, Ard JD, de Jesus JM, Houston Miller N, Hubbard VS, et al 2013 AHA/ACC guideline on lifestyle management to reduce cardiovascular risk: a report of the American College of Cardiology/American Heart Association Task Force on Practice Guidelines. J Am Coll Cardiol. 2014;63(25 Pt B):2960–84. 10.1016/j.jacc.2013.11.003 . 24239922

[6] Ashor AW, Lara J, Siervo M, Celis-Morales C, Mathers JC. Effects of exercise modalities on arterial stiffness and wave reflection: a systematic review and meta-analysis of randomized controlled trials. PLoS One. 2014;9(10):e110034 10.1371/journal.pone.0110034 25333969 PMC4198209

[7] Heffernan KS, Yoon ES, Sharman JE, Davies JE, Shih YT, Chen CH, et al Resistance exercise training reduces arterial reservoir pressure in older adults with prehypertension and hypertension. Hypertens Res. 2013;36(5):422–7. 10.1038/hr.2012.198 . 23235716

[8] Cornelissen VA, Smart NA. Exercise training for blood pressure: a systematic review and meta-analysis. J Am Heart Assoc. 2013;2(1):e004473 10.1161/jaha.112.004473 23525435 PMC3603230

[9] Kawamoto R, Kohara K, Katoh T, Kusunoki T, Ohtsuka N, Abe M, et al Effect of weight loss on central systolic blood pressure in elderly community-dwelling persons. Hypertens Res. 2014;37(10):933–8. 10.1038/hr.2014.108 . 24965169

[10] Vlachopoulos C, Aznaouridis K, Stefanadis C. Prediction of cardiovascular events and all-cause mortality with arterial stiffness: a systematic review and meta-analysis. J Am Coll Cardiol. 2010;55(13):1318–27. 10.1016/j.jacc.2009.10.061 . 20338492

[11] Heart rate variability: standards of measurement, physiological interpretation and clinical use. Task Force of the European Society of Cardiology and the North American Society of Pacing and Electrophysiology. Circulation. 1996;93(5):1043–65. . 8598068

[12] Gando Y, Yamamoto K, Murakami H, Ohmori Y, Kawakami R, Sanada K, et al Longer time spent in light physical activity is associated with reduced arterial stiffness in older adults. Hypertension. 2010;56(3):540–6. 10.1161/hypertensionaha.110.156331 . 20606102

[13] Kim J, Tanabe K, Yokoyama N, Zempo H, Kuno S. Objectively measured light-intensity lifestyle activity and sedentary time are independently associated with metabolic syndrome: a cross-sectional study of Japanese adults. Int J Behav Nutr Phys Act. 2013;10:30 10.1186/1479-5868-10-30 23452372 PMC3599104

[14] O'Donovan C, Lithander FE, Raftery T, Gormley J, Mahmud A, Hussey J. Inverse relationship between physical activity and arterial stiffness in adults with hypertension. J Phys Act Health. 2014;11(2):272–7. 10.1123/jpah.2012-0075 . 23359316

[15] Gomez-Marcos MA, Recio-Rodriguez JI, Patino-Alonso MC, Agudo-Conde C, Lasaosa-Medina L, Rodriguez-Sanchez E, et al Relationship between objectively measured physical activity and vascular structure and function in adults. Atherosclerosis. 2014;234(2):366–72. 10.1016/j.atherosclerosis.2014.02.028 . 24742874

[16] Andersson C, Lyass A, Larson MG, Spartano NL, Vita JA, Benjamin EJ, et al Physical activity measured by accelerometry and its associations with cardiac structure and vascular function in young and middle-aged adults. J Am Heart Assoc. 2015;4(3):e001528 10.1161/jaha.114.001528 25792127 PMC4392434

[17] Choi L, Liu Z, Matthews CE, Buchowski MS. Validation of accelerometer wear and nonwear time classification algorithm. Med Sci Sports Exerc. 2011;43(2):357–64. 10.1249/MSS.0b013e3181ed61a3 20581716 PMC3184184

[18] Freedson PS, Melanson E, Sirard J. Calibration of the Computer Science and Applications, Inc. accelerometer. Med Sci Sports Exerc. 1998;30(5):777–81. . 9588623 10.1097/00005768-199805000-00021

[19] Van Bortel LM, Duprez D, Starmans-Kool MJ, Safar ME, Giannattasio C, Cockcroft J, et al Clinical applications of arterial stiffness, Task Force III: recommendations for user procedures. Am J Hypertens. 2002;15(5):445–52. . 12022247 10.1016/s0895-7061(01)02326-3

[20] Camm AJ, Bigger JT Jr., Breithardt G, Cerutti S, Cohen RJ, Coulmen P, et al Task Force of the European Society of Cardiology and the North American Society of Pacing and Electrophysiology. Heart rate variability: standards of measurement, physiological interpretation and clinical use. Circulation. 1996;93(5):1043–65. 8598068

[21] Ellis KJ. Human body composition: in vivo methods. Physiol Rev. 2000;80(2):649–80. . 10747204 10.1152/physrev.2000.80.2.649

[22] Hall ME, do Carmo JM, da Silva AA, Juncos LA, Wang Z, Hall JE. Obesity, hypertension, and chronic kidney disease. Int J Nephrol Renovasc Dis. 2014;7:75–88. 10.2147/ijnrd.s39739 24600241 PMC3933708

[23] Koster A, Caserotti P, Patel KV, Matthews CE, Berrigan D, Van Domelen DR, et al Association of sedentary time with mortality independent of moderate to vigorous physical activity. PLoS One. 2012;7(6):e37696 10.1371/journal.pone.0037696 22719846 PMC3374810

[24] Hamilton MT, Hamilton DG, Zderic TW. Role of low energy expenditure and sitting in obesity, metabolic syndrome, type 2 diabetes, and cardiovascular disease. Diabetes. 2007;56(11):2655–67. 10.2337/db07-0882 . 17827399

[25] Green AN, McGrath R, Martinez V, Taylor K, Paul DR, Vella CA. Associations of objectively measured sedentary behavior, light activity, and markers of cardiometabolic health in young women. Eur J Appl Physiol. 2014;114(5):907–19. 10.1007/s00421-014-2822-0 . 24463602

[26] Healy GN, Wijndaele K, Dunstan DW, Shaw JE, Salmon J, Zimmet PZ, et al Objectively measured sedentary time, physical activity, and metabolic risk: the Australian Diabetes, Obesity and Lifestyle Study (AusDiab). Diabetes Care. 2008;31(2):369–71. 10.2337/dc07-1795 . 18000181

[27] Chobanian AV, Bakris GL, Black HR, Cushman WC, Green LA, Izzo JL Jr., et al The Seventh Report of the Joint National Committee on Prevention, Detection, Evaluation, and Treatment of High Blood Pressure: the JNC 7 report. JAMA. 2003;289(19):2560–72. 10.1001/jama.289.19.2560 . 12748199

[28] Williams B, Lacy PS, Thom SM, Cruickshank K, Stanton A, Collier D, et al Differential impact of blood pressure-lowering drugs on central aortic pressure and clinical outcomes: principal results of the Conduit Artery Function Evaluation (CAFE) study. Circulation. 2006;113(9):1213–25. 10.1161/circulationaha.105.595496 . 16476843

[29] Manisty CH, Hughes AD. Meta-analysis of the comparative effects of different classes of antihypertensive agents on brachial and central systolic blood pressure, and augmentation index. Br J Clin Pharmacol. 2013;75(1):79–92. 10.1111/j.1365-2125.2012.04342.x 22625662 PMC3555048

[30] Trudeau L. Central blood pressure as an index of antihypertensive control: determinants and potential value. Can J Cardiol. 2014;30(5 Suppl):S23–8. 10.1016/j.cjca.2014.03.010 . 24750979

[31] Joyner MJ, Limberg JK. Blood pressure regulation: every adaptation is an integration? Eur J Appl Physiol. 2014;114(3):445–50. 10.1007/s00421-013-2636-5 23558925 PMC3760992

[32] Roman MJ, Devereux RB, Kizer JR, Lee ET, Galloway JM, Ali T, et al Central pressure more strongly relates to vascular disease and outcome than does brachial pressure: the Strong Heart Study. Hypertension. 2007;50(1):197–203. 10.1161/hypertensionaha.107.089078 . 17485598

[33] Luke A, Dugas LR, Durazo-Arvizu RA, Cao G, Cooper RS. Assessing physical activity and its relationship to cardiovascular risk factors: NHANES 2003–2006. BMC Public Health. 2011;11:387 10.1186/1471-2458-11-387 21612597 PMC3123595

[34] Fitzgerald JD, Johnson L, Hire DG, Ambrosius WT, Anton SD, Dodson JA, et al Association of objectively measured physical activity with cardiovascular risk in mobility-limited older adults. J Am Heart Assoc. 2015;4(2). 10.1161/jaha.114.001288 25696062 PMC4345863

[35] Horta BL, Schaan BD, Bielemann RM, Vianna CA, Gigante DP, Barros FC, et al Objectively measured physical activity and sedentary-time are associated with arterial stiffness in Brazilian young adults. Atherosclerosis. 2015;243(1):148–54. 10.1016/j.atherosclerosis.2015.09.005 . 26386211 PMC4678284

[36] Ciolac EG, Bocchi EA, Bortolotto LA, Carvalho VO, Greve JM, Guimaraes GV. Effects of high-intensity aerobic interval training vs. moderate exercise on hemodynamic, metabolic and neuro-humoral abnormalities of young normotensive women at high familial risk for hypertension. Hypertens Res. 2010;33(8):836–43. 10.1038/hr.2010.72 . 20448634

[37] Munk PS, Butt N, Larsen AI. High-intensity interval exercise training improves heart rate variability in patients following percutaneous coronary intervention for angina pectoris. Int J Cardiol. 2010;145(2):312–4. 10.1016/j.ijcard.2009.11.015 . 19962772

